# In Vitro Study of the Effects of Pesticide Mixtures Used in Maize Cultivation in Ecuador

**DOI:** 10.3390/toxics13070530

**Published:** 2025-06-24

**Authors:** Ana Paulina Arévalo-Jaramillo, Jackeline Elizabeth Guamán Hurtado, Gabriela Cevallos-Solorzano, Natalia Bailon-Moscoso

**Affiliations:** 1 Programa de Doctorado en Ciencias, Escuela Internacional de Doctorado (EIDUNED), Universidad Nacional de Educación a Distancia (UNED), Senda del Rey 9, 28040 Madrid, Spain; ncbailon@utpl.edu.ec; 2 Departamento de Ciencias de la Salud, Universidad Técnica Particular de Loja, San Cayetano alto s/n, Loja 1101608, Ecuador; jeguamanx@utpl.edu.ec (J.E.G.H.); gcevallos@utpl.edu.ec (G.C.-S.)

**Keywords:** pendimethalin, DNA damage, comet assay, H2AX, Ecuador

## Abstract

Ecuador, located in South America, ranks among the countries with the highest rates of pesticide use per unit of cropland. Pesticide exposure is linked to genotoxic effects and carcinogenicity. While most studies evaluating the effects of pesticides focus on the active ingredient, commercial formulations are complex mixtures of several components that may alter their toxicological profile. In this study, we analyzed four pesticides commonly used in corn cultivation, and their typical field mixtures, including the herbicides atrazine and pendimethalin, the insecticides chlorpyrifos and cypermethrin, and a fertilizer, to evaluate their genotoxic effects, oxidative status, and potential to induce cellular transformation. CHO-K1 cells were treated with subtoxic doses of these formulations. MTS, comet, micronucleus, H2AX expression, SOD and GPx activity, and wound healing assays were performed. The results showed these formulations induced genotoxicity, evidenced by the comet assay. Additionally, exposure activated cellular DNA repair mechanisms, evidenced by a 1.89- to 2.63-fold increase in H2AX expression across all treatments and mixtures after 10 h. Notably, pendimethalin was associated with signs of cellular transformation, as evidenced by a 1.4-times greater cell migration observed in the wound healing assay. These findings suggest that even at subtoxic concentrations, these pesticide formulations can cause genetic damage and potentially alter cellular control mechanisms.

## 1. Introduction

The Americas have been the largest user of pesticides among all regions, with 1.78 Mt in 2021. The most used pesticides are herbicides (64%), fungicides and bactericides (15%), and insecticides (14%) [1]. This region also applies the highest levels of pesticides per area of cropland (4.7 kg/ha). Ecuador, a country in South America, is one of the countries with the highest rates of pesticide use per area of cropland (7.78 kg/ha by 2021), 3.12 points from Brazil (10.9 kg/ha), which was the world’s largest user of pesticides in 2021 according to FAOSTAT data [1].

In Ecuador, one of the most economically important crops is corn, to which 362473 hectares are destined, and whose production was 1.6 million tons in 2022 [2]. This crop is associated with a high use of pesticides, among which the following stand out: herbicide-type pesticides, such as atrazine and pendimethalin, and insecticides such as chlorpyrifos, cypermethrin, and their mixtures. The corn crop was also associated with a greater potential risk to the health of the surrounding population according to a potential index of exposure to pesticides, PPEI [3].

Pesticide exposure has been associated with genotoxic effects, as well as with an increase the oxidative stress, followed by an adaptive response to increase antioxidant defenses and prevent adverse effects [4,5,6]. An important defense mechanism is the glutathione peroxidase and superoxide dismutase system, which protects the cell from oxidizing agents [7,8]; and DNA damage-sensing mechanisms such as ɣH2AX, which activates the repair of genetic material while maintaining its integrity [9,10].

At the population level, exposure to pesticides has been related to alterations in the functioning of different organs and systems such as irritation of the respiratory and skin systems, problems in some organs, reproduction, hormonal alteration, and birth defects in humans, affection of intellectual development, and cancer, among others [11,12,13]. In Ecuador, some studies have reported that the health of populations might be compromised by the extensive use of pesticides in plantations; farmworkers have shown genotoxicity associated with exposure to pesticides, biochemical and genotoxic effects have been reported in women indirectly exposed to pesticides, and high exposure during gestation increases the likelihood of a low birth weight and preterm delivery [14,15,16].

Agricultural practice also includes the use of fertilizers that are used to restore nutrients to the soil to improve production. Harmful effects have been reported due to an excess or accumulation of metals or nitrogen compounds, among others present in these products, related to the development of metabolic disease by altering the kidney and liver functions and inducing hematological abnormalities [17,18]. In Ecuador, 297 kg/ha of fertilizers are used in corn cultivation [19].

Generally, the studies evaluate the effects of pesticides and analyze only the active ingredients, but pesticide formulations are a mixture of ingredients. Ignoring the possible risks deriving from the interaction between the active and other ingredients of commercial products might result in the misinterpretation of their toxicological profiles [20,21,22]. Therefore, in the present study, four products widely used by farmers in corn cultivation are analyzed, as well as the mixtures usually applied in the field, that is, herbicides and insecticides and fertilizers, to evaluate their genotoxic effect, alteration of the oxidative state, and cell transformation.

## 2. Materials and Methods

### 2.1. Treatment

Cells were treated with various concentrations of pesticides according to Table 1. These pesticides were obtained from a commercial products available in the local market, as follows: Gesaprim (Syngenta, St. Gabriel, LA, USA), containing 900 g/kg of atrazine; Gramilaq 400 (Agripac SA, Guayaquil, Ecuador), containing 400 g/L of pendimethalin; Bala 55 (Reap, Sulphur Mills LTD, Mumbai, India), containing 500 g/L of chlorpyrifos and 50 g/L of cypermethrin; and Fuerza verde (Plantgrow, GA, USA) which contains among its main components 9% nitrogen, 24% phosphorus, 27% potassium, and boron, copper, manganese, molybdenum, zinc, and iron, which together represent 0.22%. The doses evaluated took as a reference those proposed by the WHO as the maximum allowable limit of these compounds in water for human consumption [23], which are in accordance with Ecuadorian legislation considering the technical standard INEN1108 [24]. In addition to the dose indicated as the maximum permissible limit, a lower and higher dose was used in the study. The mixtures evaluated correspond to those used in the field by farmers, which include mixing products containing herbicides and mixing those containing insecticides with fertilizer.

### 2.2. Cell Culture

A CHO-K1 cell line derived from Chinese hamster ovary cells (Sigma-Aldrich, USA. 85051005, Lot: 17E029) was used. Cells were cultured in HAMF-12 (Gibco, Grand Island. NY, USA) medium supplemented with FBS 10% (Sigma, St. Louis MD, USA), 1% antibiotic-antimitotic solution (100 units/mL penicillin G, 100 μg/mL streptomycin, 0.25 μg/mL amphotericin B, Gibco, Grand Island, NY, USA), and L-glutamine (2mM, Gibco, São Paulo, Brazil), HEPES (20 mM, Gibco, Grand Island, NY, USA), sodium bicarbonate (2%, Fisher Scientific, Brisbane, Australia); and maintained at 37 °C in a humidified atmosphere containing 5% CO_2_.

### 2.3. Cell Proliferation Assay

Cell proliferation was measured using Cell Titer 96 Aqueous One Solution cell proliferation reagent (Promega, Madison, WI, USA) and by seeding 3 × 10^3^ cells/well into 96-well plates in 100 μL of medium and incubated for 24 h. Then, cells were treated with pesticides at different concentrations (Table 1) in triplicate for 24 and 48 h. Four hours before finishing the treatment, 20 μL of reagent was added to each well. The plates were then maintained at 37 °C; the absorbance of each sample was measured with a micro-plate reader (Epoch 2—BioTek, Winooski, VT, USA) at 570 nm. Cells without treatment were used as a control. The data obtained with untreated cells considered as 100% of cell proliferation [25].

### 2.4. Comet Assay

The comet assay was performed according to the method described by Bailón et al. [26]. In total, 5 × 10^3^ cells/well were seeded into 96-well plates in 100 μL of medium and incubated for 24 h. Then, cells were treated with pesticides at different concentrations according to Table 1 in duplicate for 24 h.

After the treatment, the cells were trypsinized, the cell content was placed in 1.5 mL microtubes and underwent centrifugation 10,000 rpm at 4 °C for 5 min to obtain the cell pellet. The pellet was suspended in 150 µL of 1% low-melting-point agarose (Invitrogen, Carlsband, CA, USA), and 75 µL was placed on two slides containing a layer of 1% agarose (Invitrogen, Carlsband, CA, USA), previously prepared, with a cover slip, and refrigerated for 10 min; the coverslip was removed and 130 µL of low-melting-point agarose was added. The cover slip was replaced and the slide refrigerated for 10 min. After solidification of the agarose layer, the slides were immersed in lysis solution (10% DMSO (Fisher Bioreagents, Waltham, MA, USA), 1% Triton X-100 (Fisher Bioreagents, USA), 2.5 M NaCl (Merck, Darmstadt, Germany), 100 mM EDTA (Sigma-Aldrich, St. Louis, MI, USA), and 10 mM Tris (Invitrogen, Waltham, MA, USA), at pH 10 and 4 °C, protected from light for at least 1 h. After lysis, the slides were placed in electrophoresis buffer (1 mM NaOH, 300 mM EDTA pH 13, Sigma-Aldrich, St. Louis, MI, USA) for 20 min at room temperature. Next, electrophoresis was carried out on a horizontal electrophoresis platform with cooling for 20 min at 300 mA, 25 V. The slides were neutralized with 0.4M Tris-Base buffer at pH 7.5 (previously stored at 4 °C) and fixed with cold ethanol (Sigma-Aldrich, St. Louis, MI, USA). Staining was performed with ethidium bromide (Sigma-Aldrich, USA), (30 µg/mL) for 1 min. Each slide was analyzed using a fluorescence microscope (Zeiss Axioskos 2 plus, Oberkochen, Germany) with a 40X objective. For each plate, 100 comets were analyzed using the Comet Assay IV v4.3 software system, determining tail length, tail intensity, and tail moment. Methyl methane sulfonate at 10 µM (Fisher Scientific) was used as a positive control.

### 2.5. Micronucleus Assay

The micronucleus assay was performed according to the method described by Bailón et al. [26] with modifications. In total, 2 × 10^5^ cells/well were seeded into 6-well plates contained a coverslip, in 2000 μL of medium and incubated for 24 h. Then, cells were treated with pesticides at different concentrations according to Table 1, in duplicate for 24 h. The cells were washed with PBS (Sigma-Aldrich, St. Louis, USA), and then fixed with a 1:1 methanol-PBS solution by 2 min and methanol (Merck, Germany) by 10 min. The slides were dried at room temperature, next stained with 0.003% of acridine orange (Santa Cruz Biotechnology, Dallas, TX, USA) in Sorensen buffer for 10 min and a final wash with Sorensen pH 6.8 buffer. The micronuclei (MN), nuclear buds (NBuds), Notched cells, binucleated cells (BN), and nucleoplasmic bridges (NPBs) were analyzed in 2000 cells from each slide, according to the criteria established by Fenech 2007, and the nuclear division index (NDI) was calculated for each experimental using the formula NDI = [(N1) + 2(N2) + 3(N3+)]/N, where N1–N3+ represent the number of cells with one to three or more nuclei, and N is the total number of viable cells scored. The analysis was performed using a fluorescence microscope (Zeiss Axioskos 2 plus, Germany) with a magnification of × 100. The Mitomycin C at 0.5 µg/mL (Sigma-Aldrich, St. Louis, MI, USA) was used as positive control.

### 2.6. H2AX Expression

To evaluate the expression of H2AX, RNA was extracted from approx. 2 × 10^6^ cells after treatment with pesticides for 3 and 10 h. Total RNA was isolated from cells using the RNA extraction kit (Cat. R1035, Zymo-Research, Tustin, CA, USA) according to the manufacturer’s instructions. RNA purity and concentration were determined using a NanoDrop2000 (Thermo-Scientific, USA). First-strand cDNAs were synthesized using the SuperScript VILO cDNA Synthesis kit (Invitrogen, USA) according to the manufacturer’s instructions. Quantification of gene expression of the H2AX (Cg04435464_s1) variant was performed in duplicate using TaqMan Gene Expression Assays technology (Applied Biosystems, Pleasanton, CA, USA) for the CHO-K1 cell line, using GADPH (Cg04424038_gH) as an endogenous reference. Experiments were performed in duplicate for each sample and the Ct data of each sample was determined using default threshold settings. Relative quantification of mRNA expression was calculated with the 2^−ΔΔCt^ method using a 7500 Fast Real-Tim PCR System (Applied Biosystems, Singapore).

### 2.7. Antioxidant Enzyme Activities

To evaluate the enzyme activity, assay kits for SOD and GPx were used according to the manufacturer’s instructions (Superoxide Dismutase, SOD, Activity, Assay Kit of Sigma, St. Louis, MO, USA, and Glutathione Peroxidase Assay Kit, Colorimetric, of Abcam ab102530, Waltham, MA, USA). Briefly, the cells were seeded in 6-well plates at 5 × 10^5^ cells/well in 2000 μL of medium and incubated for 24 h. Then, cells were treated with pesticides in duplicate and exposed to pesticides for 4 and 24 h at 37 °C. The cells were harvested by trypsinization and normalized with a protein concentration using the Bradford assay (Bradford Reagent, St. Louis, MI, USA), before to measure in a multiple plate reader at 450 nm for SOD and 340 nm for GPx [27,28]. Sodium azide at 10 mM (Sigma-Aldrich, St. Louis, MI, USA) and 4-hidroxinonenal at 0.12 mM (Sigma-Aldrich, St. Louis, MI, USA) were used as controls for their effect as inhibitors of enzymatic activity for SOD and GPx, respectively.

### 2.8. Scratch Wound Healing Assay

A scratch wound healing assay was performed to assess the effect of the pesticides on cell migration. Cells were seeded in six-well plates until 90–100% confluent. Then, the cell-coated surface was scratched with a 200 μL pipette tip in a single stripe and washed two times with PBS (Sigma-Aldrich, St. Louis, MI, USA). Then, cells were treated with pesticides at different concentrations according to Table 1 for 24 h at 37 °C and 5% CO_2_. The cell migration and wound closure over 24 h were captured as a time lapse by the imaging system of the Axio observer 7 microscope (Zeiss, Germany). The images were analyzed using Image J software v1.54 with the plugin Migration to measure the migration [29].

### 2.9. Statistical Analysis

Three independent experiments were performed in duplicate. Data are presented as the mean ± SEM. Comparisons between multiple groups were analyzed using a one-way ANOVA followed by Dunnett’s post hoc test, using GraphPrism program, version 8.0 for Windows. *p* < 0.05 was considered to indicate a statistically significant difference.

## 3. Results

**Figure 1:**
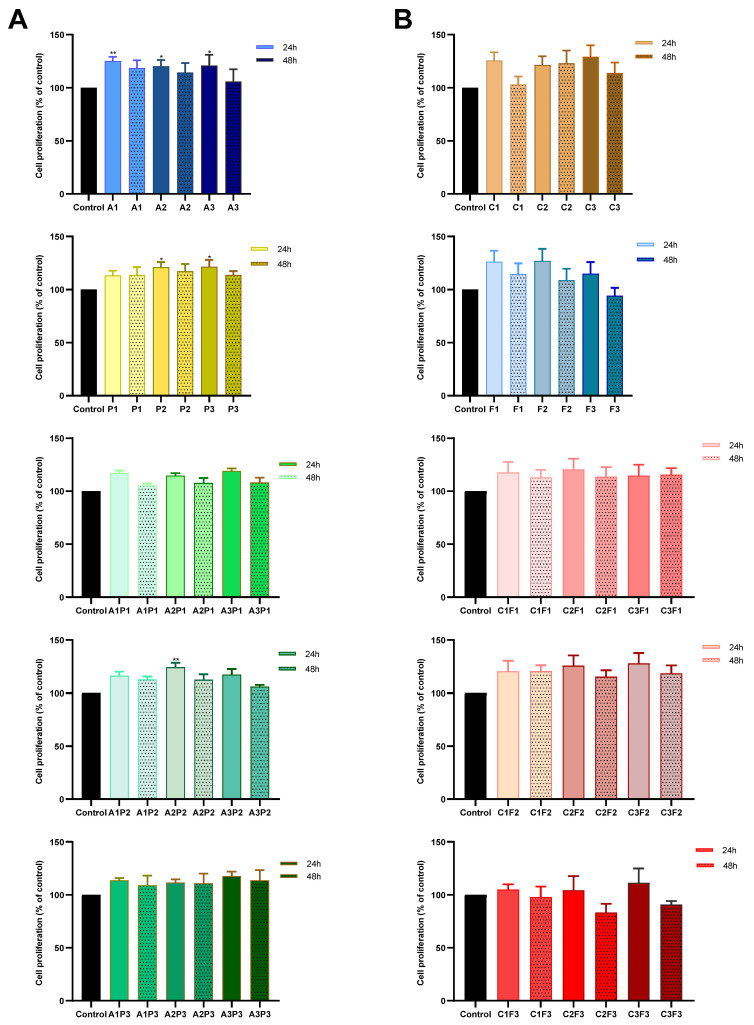
Mean cell proliferation assay performed on CHO-K1 cells. The bars represent the mean values ± SEM at 24 and 48h exposed to (**A**) herbicides: atrazine, pendimethalin, and their mixtures, and (**B**) insecticides: chlorpyrifos/cypermethrin and fertilizer, and their mixtures. Significant statistical difference as compared to the negative control using ANOVA followed by Dunnett’s test * (*p* < 0.05) ** (*p* < 0.01).

Our results showed that most of the evaluated doses, indicated in Table 1, did not significantly affect the cell proliferation of most compounds compared to the control, at both 24 and 48 h of exposure—except the herbicides atrazine and pendimethalin and their mixture, which induced a significant increase in cell proliferation at 24 h. This effect was consistently observed across all atrazine doses (A1, A2, and A3), the higher pendimethalin doses (P2 and P3), and the A2P2 mixture (Figure 1).

After evaluating the effect of treatments on cell proliferation, in which cell growth was observed with some pesticides, genotoxicity assays were performed to evaluate their effect on the genetic material.

**Figure 2:**
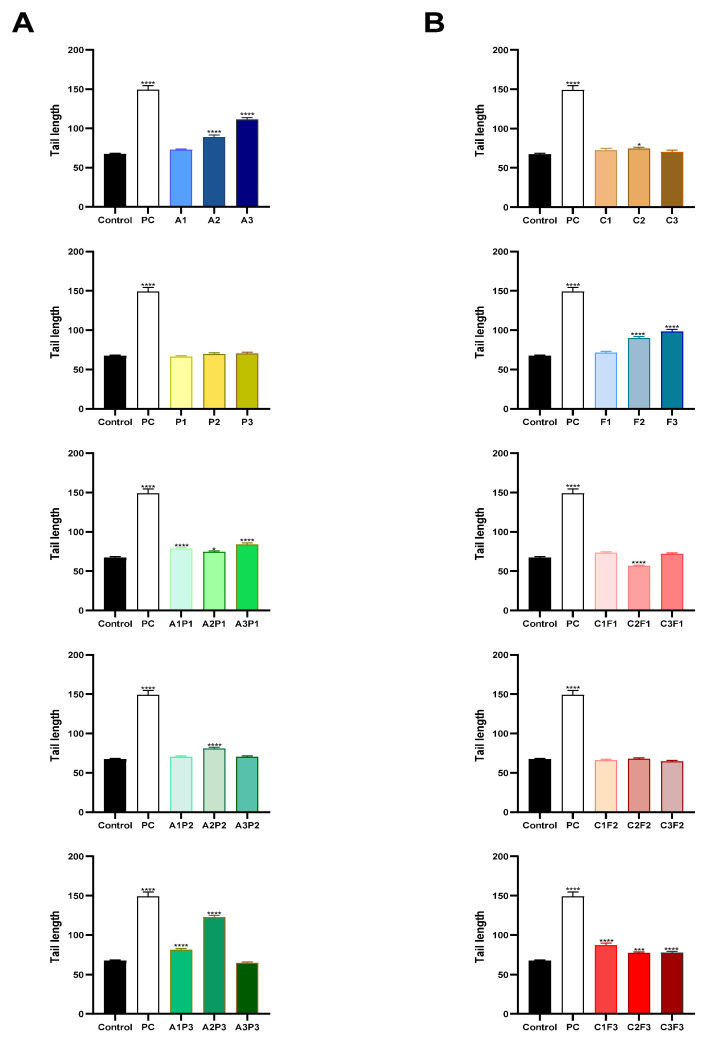
Comet assay of DNA damage in CHO-K1 cells exposed for 24 h to pesticides. Tail length in (**A**) herbicides: atrazine, pendimethalin, and their mixtures, and (**B**) insecticides: chlorpyrifos/cypermethrin and fertilizer, and their mixtures. PC = positive control methyl methane sulfonate 10 µM. The data are presented as the mean ± SEM. Significant statistical difference as compared to the negative control using ANOVA followed by Dunnett’s test * (*p* < 0.05) *** (*p* < 0.001) **** (*p* < 0.0001).

The comet assay was used to analyze the genotoxicity of pesticides at various concentrations in CHO-K1 cells. A significant level of DNA damage, as measured by tail length, was identified with herbicides, the higher doses of atrazine A2 and A3, and combinations that included low concentrations of atrazine and pendimethalin, even though pendimethalin has not shown a significant difference alone. In the same way, a significant level of DNA damage was identified with the insecticides chlorpyrifos/cypermethrin at C2 and the higher doses of fertilizer F2 and F3, as well as mixtures that included F3 (Figure 2). Data about the tail intensity and tail moment are shown in the Supplementary Material, Figures S1 and S2, respectively.

The MN assay measures the damage induced to the chromosomes and mitotic apparatus; the treatments evaluated did not induce MN. For methodological reasons, for this trial and subsequent trials, only the reference doses, indicated as the maximum permissible limit, for each pesticide are evaluated. An increased frequency of PNB was found in the treatment that included a mixture of herbicides A2P2 (Table 2); this mixture also showed DNA damage in the comet assay by tail length.

**Figure 3:**
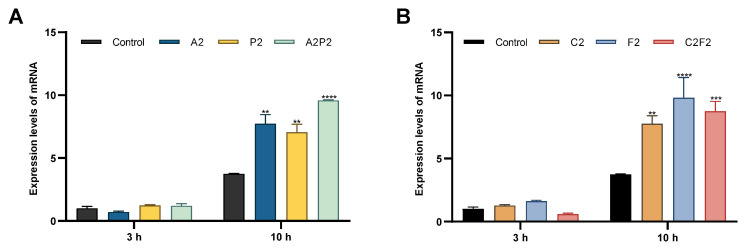
Gene expression analysis of H2AX. Gene expression levels of H2AX measured by qRT-PCR in CHO-K1 cells after 1, 3, and 10 h exposure to atrazine and pendimethalin (**A**) and chlorpyrifos/cypermethrin and fertilizer (**B**). Data show mean ± SEM, Significant statistical difference as compared to the negative control using ANOVA followed by Dunnett’s test **(*p* < 0.01) *** (*p* < 0.001) **** (*p* < 0.0001).

Another genotoxicity test used was the expression of the DNA repair-related gene H2AX examined by real-time PCR. The mRNA levels of H2AX increased significantly after 10 h of exposure in all treatments: the herbicides atrazine A2 and pendimethalin P2, their mixture A2P2; and the insecticides chlorpyrifos/cypermethrin C2, fertilizer F2, and their mixture C2F2 (Figure 3). The treatments A2, A2P2, C2, and F2 that show an increase in H2AX expression also reveal damage to the genetic material in the comet assay.

**Figure 4:**
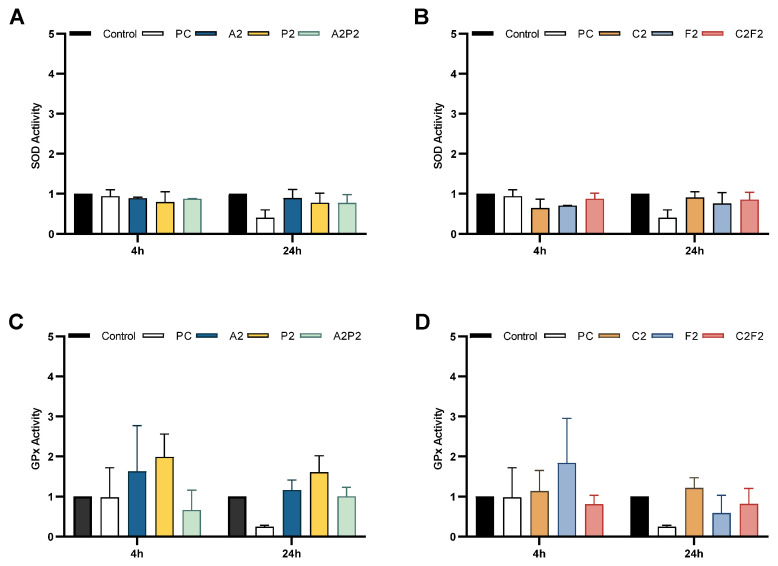
Effects of pesticides on the activity of SOD and GPx in CHO-K1. Cells were exposed for 4 h and 24 h to atrazine, pendimethalin, and their mixture (**A,C**); and chlorpyrifos/cypermethrin, fertilizer, and their mixture (**B,D**). PC = positive control, inhibitor of enzymatic activity: sodium azide 10 mM for SOD and 4-hidroxinonenal 0.12 mM for GPx. Data show the mean ± SEM.

The oxidative status in CHO-K1 cells upon exposure to pesticides was investigated by measuring the activities of the main enzymes involved in the control of ROS level. Figure 4 shows the enzymatic activity after 4 and 24 h of treatment with pesticides and their mixtures; no significant differences were observed.

**Figure 5:**
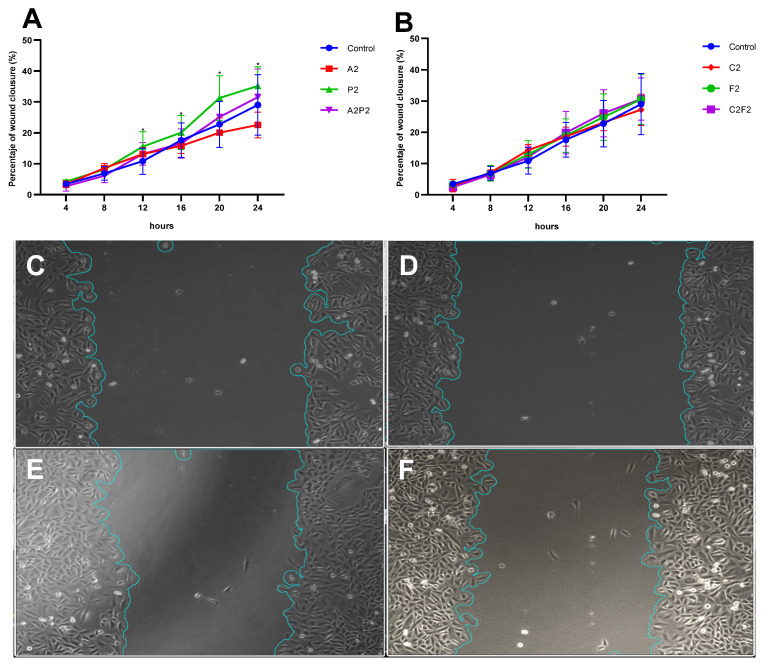
Effects of pesticides on the wound healing assay in CHO-K1. Cells were exposed for 24 h to atrazine, pendimethalin and their mixture (**A**); and chlorpyrifos/cypermethrin, fertilizer, and their mixture (**B**). Data show the mean ± SEM, Significant statistical difference as compared to the negative control one-way ANOVA with a Friedman test for multiple comparison * (*p* < 0.05). Scratch wound healing assay of control and pendimethalin at 0 h (**C**,**D**) and 24 h (**E**,**F**), respectively.

Finally, the effect of pesticides on a possible cell transformation was evaluated by the wound closure assay, which analyzed cell migration. We observed a significant increase in the percentage of wound closure in cells treated with P2 compared to the control, evaluated during 24 h (Figure 5).

## 4. Discussion

Ecuador is one of the countries with the highest rates for pesticide use per area of cropland [1], which has important consequences for the health of the population, considering that 28% of Ecuador’s population dwell in areas with a high pesticide application rate. At the national level, the province of Loja presents one of the highest rates of pesticide application with 47.4 kg/ha per year [30], a situation that is worrying considering the operational deficiencies at the time of pesticide application in the field. As Ecuador is a country whose economy depends on agricultural production, it is important that this issue be addressed from different perspectives to improve the sources of information and strategies that can be implemented for the health care of the population. In this study, we evaluate the genotoxic effect of four pesticides widely used by farmers in corn cultivation in southern Ecuador, considering doses that according to Ecuadorian regulations constitute the maximum limits allowed in water for human consumption.

In our study, we found that cells exposed to the herbicides atrazine (0.01, 0.1, 1 µg/mL) and pendimethalin (0.02, 0.2 µg/mL) and their mixture that included 0.1 of atrazine and 0.02 of pendimethalin, significantly increased cell proliferation compared to the control at 24 h of treatment. Previous studies in which the effects of these pesticides have been evaluated show different results; for example, Kmetič et al. [31] in their study showed that atrazine exposure led to a decrease in cell proliferation in the CHO-K1 cell line after 72 h, using concentrations from 10 to 160 µg/mL. The same effect was reported in normal human fibroblasts after exposure (10–300 µg/L) for 48 h, observing a decreased cell proliferation in response to atrazine without significant changes in DNA fragmentation or caspase activity, indicating a specific effect on cell growth within this timeframe [32].

Other studies such as Tian et al. [33], in concordance with us, report that exposure to atrazine at 0.01 mM and 0.1 mM for 48–72 h increased in the proliferation of H22 cells, an effect that was associated with the upregulation of proteins like cyclin-D1, VEGF, MMP2, Stat3, and C-myc, and the downregulation of p53 supported increased cell viability, suggesting a promotion of cell proliferation and invasion by atrazine. Likewise, in a study carried out in a primary culture of melanocytes, exposure to atrazine at 0.1 and 10 µM may lead to decreased apoptosis and increased cell survival, potentially affecting cell viability in primary skin cultures, which would be mediated by the anti-apoptotic marker BCL-2 that was notably increased in exposed cells [34].

Studies with pendimethalin also indicate diverse results; cell viability was decreased in human umbilical vein endothelial cells, HUVECs, treated with 50 and 100 µM pendimethalin, and the proportion of apoptotic and necrotic cells was increased [35], but on the other hand, the effects of pendimethalin in A549 human lung carcinoma cells after 24 h’ exposure with 100 μM altered the apoptosis-related gene expression and significantly downregulated BAX, P53, and CAS3, suppressing apoptosis and allowing cancer cells to grow and proliferate [36]. These findings suggest that the herbicides atrazine and pendimethalin, at sublethal concentrations, alone or in a mixture have effects on cellular viability and function, highlighting their role as a potential regulator in the cell cycle in different cell types.

In our study, genotoxicity assays showed a significant level of DNA damage as measured by the comet assay identified with the herbicides, insecticides, and fertilizer, and an increased frequency of NPBs was found in the treatment that included a mixture of the herbicides atrazine and pendimethalin. Also, we observed that the levels of H2AX expression increased significantly as an earlier response to treatment with insecticides and increased in all treatments of pesticides after 10 h of exposure.

In the same way, previous reports about these pesticides have shown their genotoxic effect. The herbicide atrazine has shown the potential to trigger DNA double-strand breaks and activate the DNA-damage response pathway in MCF-10A cells, in which it is observed to increase the expression of tumor necrosis factor receptor-1 (TNFR1), phosphorylated Rad17, elevate the levels of H2AX phosphorylation (γH2AX) and the formation of γH2AX foci, and increase the levels of the DNA-damage checkpoint proteins ATR, ATRIP, and phospho-Chk1 [37]. The herbicide pendimethalin has a reported genotoxic effect in vitro [38] and in vivo through the comet assay, in addition to leading to changes in oxidative stress markers, increased protein carbonylation and lipid peroxidation, decreased levels of antioxidants like GSH, SOD, CAT, and GST, and activation of the intrinsic apoptotic pathway [39].

The insecticides chlorpyrifos and cypermethrin also have a previously reported genotoxic effect; in human leukocytes, exposure to these compounds increased the micronucleus frequency, numerical chromosomal instabilities, and apoptotic cells [40]; and in HeLa, HEK293, and HepG2 cells, these insecticides increased the number of single- and double-strand DNA breaks, the percentage of ɣH2AX, DNA fragmentation, and intracellular ROS [41,42,43]. Fertilizers also have reported deleterious impacts on biomarkers of the oxidative stress status, with a significant decrease in antioxidant enzymes SOD, CAT, and GPx, possibly due to an inhibitory effect generated by the heavy metals present in these compounds [17,18]. Our results allow us to identify a genotoxic effect of formulations that contain the herbicides atrazine and pendimethalin and their mixtures, as well as formulation that contain the insecticides chlorpyrifos and cypermethrin and a fertilizer formulation, evidencing damage to the genetic material and a cellular response to damage through the expression of H2AX for the activation of repair mechanisms [44].

As mentioned above, in some studies, exposure to pesticides shows increased intracellular ROS levels and changes in the levels of antioxidants system activity [17,39,42,43]. We observed an non-significant increased activity of GPx mediated by herbicides and chlorpyrifos/cypermethrin, as an earlier response and effective for combating oxidative stress [9], in this case related to nonradical species [45]; we also observed a decrease in SOD activity, especially with fertilizer as an inhibitory effect by its composition [17,18]. The elevation of GPx activity as well as the decrease in SOD could confirm the occurrence of oxidative stress within the cells [43].

The cell transformation, as an effect of pesticide exposure, was evaluated by cell migration using the wound closure assay, observing a significant increase in the percentage of wound closure in cells treated with pendimethalin at 0.02 µg/mL. Unlike what was found in this work, other studies showed that pendimethalin at 25, 50, and 100 μM decreased the wound closure by 59%, 73%, and 91%, respectively, in human umbilical vein endothelial cells, HUVECs, indicating that pendimethalin induced a suppression of endothelial functions [35], which differs from what was found in our study, possibly due to the dose used.

A situation similar to that observed in our work has been reported for other pesticides such as malathion and its metabolites dimethyl thiophosphate and dimethyl phosphate, which present an increase in the percentage of wound closure compared to the control, which decreases as the dose of the compound increases, an effect that could be explained by the dual role of Rac1 in cell migration [46]. Rac1 activation induces cell protrusions such as lamellipodia that favor cell migration, but Rac1 activation can also recruit scaffold proteins, blocking its interaction with the actin cytoskeleton, making stronger cell–cell interactions that in turn reduce cell migration [47]. In the same way, alterations in the actin cyto-skeleton and decreased expression of the adhesion molecules E-cadherin and β-catenin in the breast cancer line MCF-7 cells at a low dose of this pesticide resulted in the loss of E-cadherin accompanied by an increase in the expression of Rho and Rac1 GTPases, which promote cytoskeleton rearrangements, migration, and invasion [48].

In our study, pendimethalin at 0.02 µg/mL shows an increase on cell migration, which could be linked to molecular changes associated with a cellular transformation, which must be studied in detail to understand the cellular mechanisms that control cell migration and its importance in metastasis.

This study, while valuable for preliminary hazard identification using the in vitro CHO-K1 cell model, acknowledges key limitations. The CHO-K1 cell line, being rodent-derived, may not fully replicate human physiological responses, and the pesticide concentrations used, though based on regulatory limits, may not reflect in vivo bioavailability in human organs. Therefore, direct extrapolation of these findings to human health risks should be performed with caution. Future research is crucial to complement these findings, including in vivo studies, the use of human-derived cell lines to assess organ-specific effects, the incorporation of ɣH2AX analysis as a DNA-damage biomarker, and the extension of exposure time in the wound healing assay, for a deeper understanding.

This study provides valuable insights into the genotoxic and cell-transforming effects of the analyzed commercial pesticides at regulatory limits, highlighting the impacts they produce both individually and in mixtures.

## 5. Conclusions

In this work, it is evident that these formulations of herbicides, insecticides, fertilizer, and their mixtures at subtoxic doses cause genotoxicity in the CHO-K1 cell line, as a response to exposure to pesticides. It was also possible to identify that this exposure activates the cellular antioxidant enzymatic response and repair mechanisms of DNA and to highlight the effect of the herbicide pendimethalin, which could be related to a cellular transformation.

## Figures and Tables

**Table 1:** Pesticides used: type and dose.

Type	Pesticide	Dose (µg/mL)		
Herbicide	Atrazine (A)	0.01 A1	0.1 A2	1 A3
	Pendimethalin (P)	0.002 P1	0.02 P2	0.2 P3
Insecticide	Chlorpyrifos/cypermethrin ^+^ (C)	0.003 C1	0.03 C2	0.3 C3
Fertilizer	Phosphorus * (F)	1 F1	10 F2	100 F3

+ The concentration of chlorpyrifos in the product was considered as a reference for the calculations. * The percentage of P_2_O_5_ in the product was considered as a reference for the calculations.

**Table 2:** Frequency of MN and nuclear abnormalities.

Treatment	MN	NBUDs	NOTCH	PNB	NDI
A2	33.67 ± 3.48	222.00 ± 35.82	8.66 ± 1.97	6.83 ± 0.98	1.97 ± 0.02
P2	25.17 ± 2.85	180.80 ± 40.01	8.33 ± 2.77	7.83 ± 0.54	1.98 ± 0.02
A2P2	27.83 ± 1.88	183.80 ± 15.21	8.16 ± 2.12	9.00 ± 0.68 **	1.99 ± 0.01
C2	25.33 ± 3.42	172.50 ± 16.36	5.16 ± 1.16	5.00 ± 0.93	1.93 ± 0.02
F2	24.83 ± 3.43	132.00 ± 21.19	5.50 ± 1.31	4.66 ± 0.55	1.96 ± 0.03
C2F2	25.83 ± 3.04	129.20 ± 21.11	4.50 ± 1.14	5.66 ± 0.84	1.95 ± 0.01
PC	35.00 ± 3.21 *	198.00 ± 48.51	5.33 ± 2.33	4.33 ± 0.33	1.90 ± 0.04
Control	26.67 ± 1.38	210.80 ± 50.22	5.00 ± 0.85	5.16 ± 0.70	1.92 ± 0.04

PC = positive control Mitomycin C 0.5 µg/mL. Results expressed as mean ± SEM. * Significant statistical difference as compared to the negative control using a *t* test (*p* < 0.05) ** (*p* < 0.01).

## Data Availability

Data is included in the article. Further inquiries can be directed to the corresponding author.

## Supplementary Materials

The following supporting information can be downloaded at https://www.mdpi.com/article/10.3390/toxics13070530/s1, Figure S1: Comet assay: DNA damage (tail intensity) in CHO-K1 cells exposed for 24 h to pesticides; Figure S2: Comet assay: DNA damage (tail moment) in CHO-K1 cells exposed for 24 h to pesticides.

## Abbreviations

The following abbreviations are used in this manuscript:

A	Atrazine
P	Pendimethalin
C	Chlorpyrifos/cypermethrin
F	Fertilizer
MTS	[3-(4,5-dimethylthiazol-2-yl)-5-(3-carboxymethoxyphenyl)-2-(4-sulfophenyl)-2H-tetrazolium, inner salt; MTS]
SOD	Superoxide dismutase
GPx	Glutathione peroxidase
H2AX	H2A.X variant histone
MN	Micronuclei
NPBs	Nucleoplasmic bridges
