# Supplementary material for: In Vitro Study of the Effects of Pesticide Mixtures Used in Maize Cultivation in Ecuador
Source: Toxics. 2025 Jun 24;13(7):530. doi: 10.3390/toxics13070530 (PMC12300072; doi:10.3390/toxics13070530)
Supplement: Supplementary file 1 [file toxics-13-00530-s001.zip › toxics-3674344-supplementary.pdf]

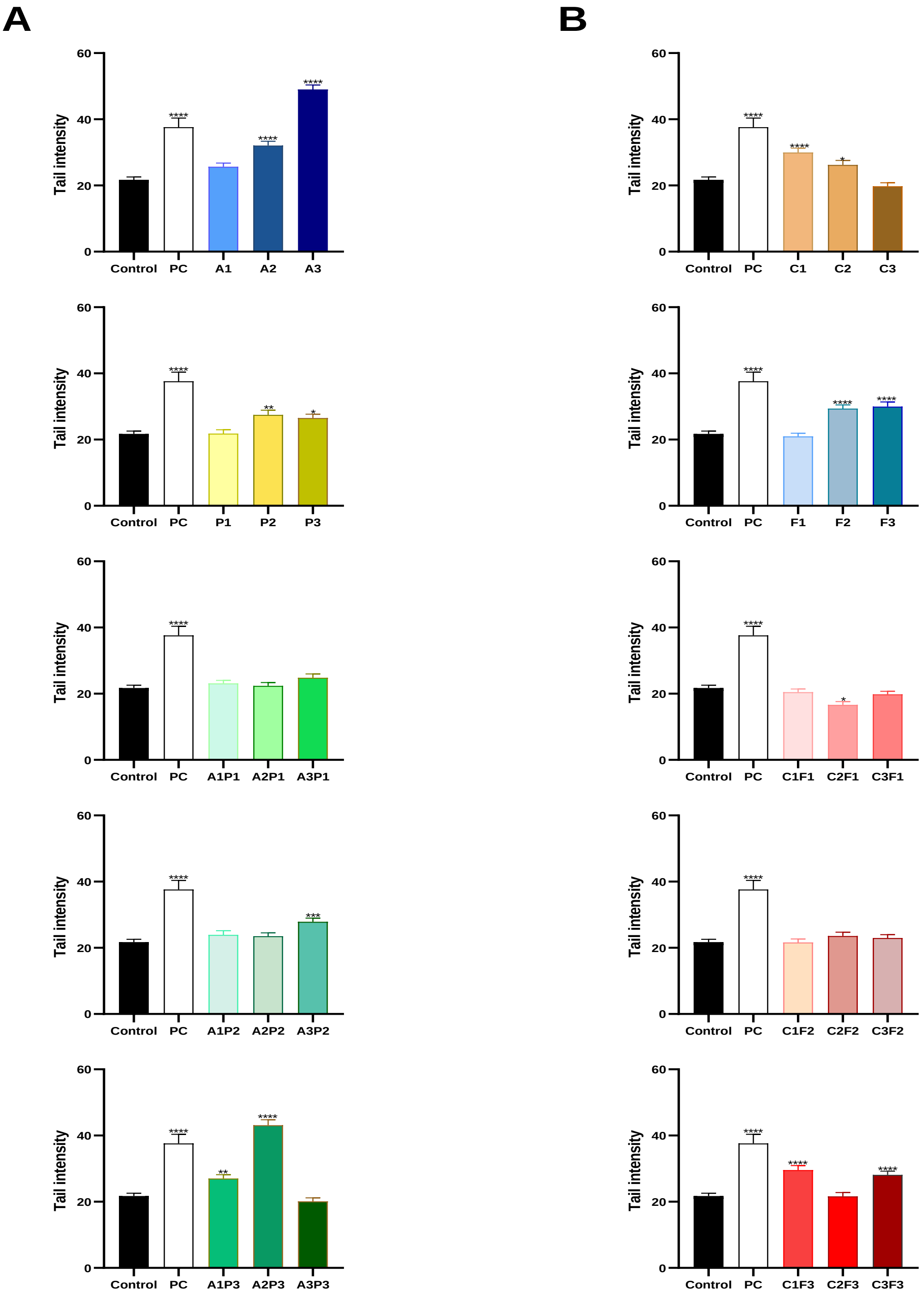

Figure S1. Comet assay: DNA damage (tail intensity) in CHO-K1 cells exposed 24 hours to pesticides. Figure shows tail intensity in (A) herbicides: atrazine, pendimethalin and their mixtures, and (B) insecticides: chlorpyrifos/cypermethrin, and fertilizer and their mixtures. PC= positive control methyl methanesulfonate 10 μM. The data are presented as the mean ± SEM. *Significant statistical difference as compared to the negative control using ANOVA followed by Dunnett's test ($p < 0.05$) **($p < 0.01$) ***($p < 0.001$) ****($p < 0.0001$)

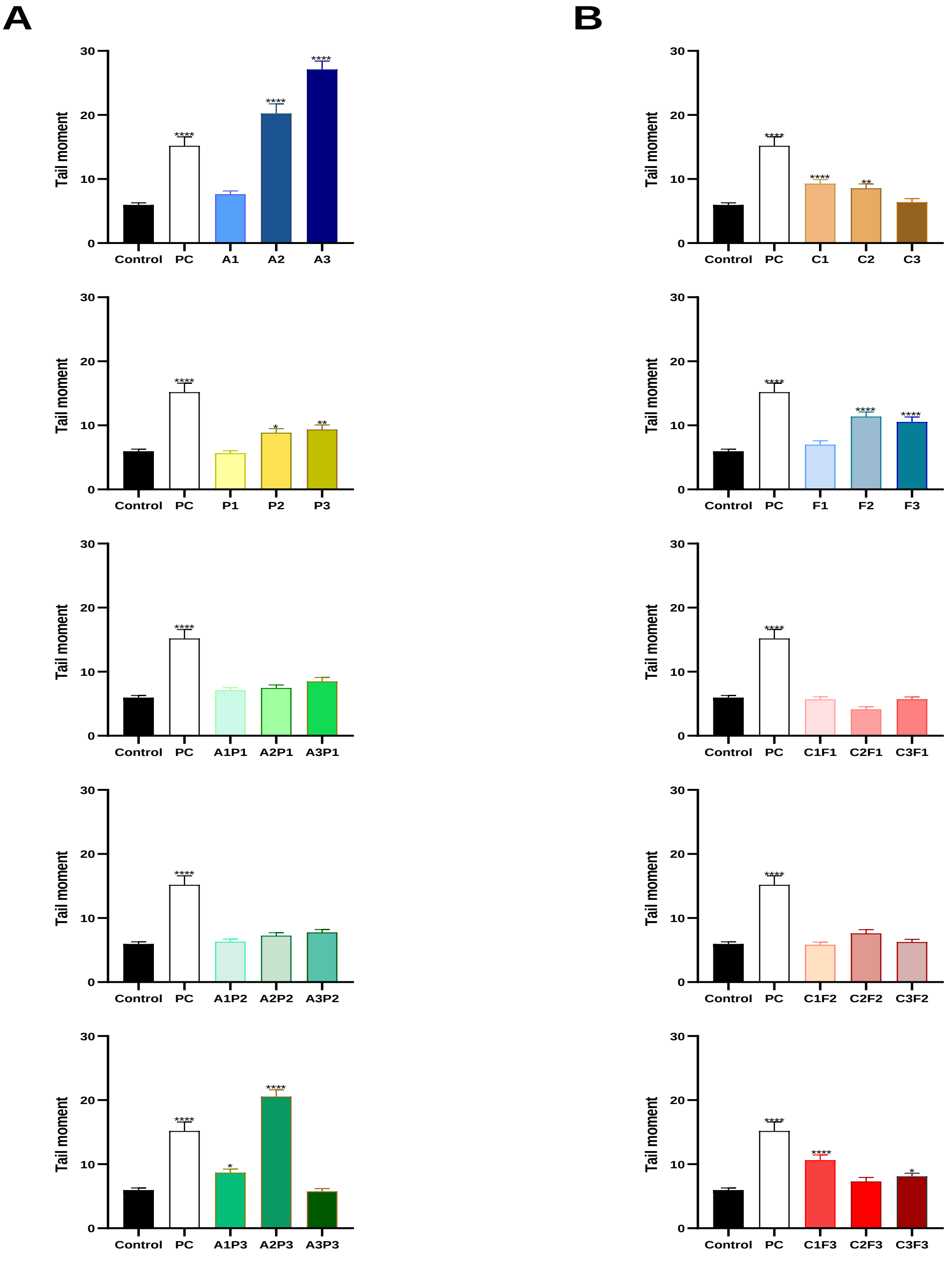

Figure S2. Comet assay: DNA damage (tail moment) in CHO-K1 cells exposed 24 hours to pesticides. Figure shows tail moment in (A) herbicides: atrazine, pendimethalin and their mixtures, and (B) insecticides: chlorpyrifos/cypermethrin, and fertilizer and their mixtures. PC= positive control methyl methanesulfonate 10 μM. The data are presented as the mean ± SEM. *Significant statistical difference as compared to the negative control using ANOVA followed by Dunnett's test ($p < 0.05$) **($p < 0.01$) ***($p < 0.001$) ****($p < 0.0001$)
